# Impact of insecticide resistance on malaria vector competence: a literature review

**DOI:** 10.1186/s12936-023-04444-2

**Published:** 2023-01-17

**Authors:** Pierre Fongho Suh, Emmanuel Elanga-Ndille, Magellan Tchouakui, Maurice Marcel Sandeu, Darus Tagne, Charles Wondji, Cyrille Ndo

**Affiliations:** 1 Department of Parasitology and Microbiology, Centre for Research in Infectious Diseases, P.O. Box 13591, Yaoundé, Cameroon; 2 Faculty of Sciences, University of Yaoundé I, P.O. Box 837, Yaoundé, Cameroon; 3 Department of Medical Entomology, Centre for Research in Infectious Diseases, P.O. Box 13591, Yaoundé, Cameroon; 4 Department of Microbiology and Infectious Diseases, School of Veterinary Medicine and Sciences, University of Ngaoundéré, P.O. Box 454, Ngaoundéré, Cameroon; 5 Faculty of Sciences, University of Douala, P.O. Box 24157, Douala, Cameroon; 6 Vector Biology Department, Liverpool School of Tropical Medicine, Pembroke Place, Liverpool, L3 5QA UK; 7 Department of Biological Sciences, Faculty of Medicine and Pharmaceutical Sciences, University of Douala, P.O. Box 24157, Douala, Cameroon

**Keywords:** *Plasmodium*, *Anopheles*, Insecticide resistance, Vector competence

## Abstract

Since its first report in *Anopheles* mosquitoes in 1950s, insecticide resistance has spread very fast to most sub-Saharan African malaria-endemic countries, where it is predicted to seriously jeopardize the success of vector control efforts, leading to rebound of disease cases. Supported mainly by four mechanisms (metabolic resistance, target site resistance, cuticular resistance, and behavioural resistance), this phenomenon is associated with intrinsic changes in the resistant insect vectors that could influence development of invading *Plasmodium* parasites. A literature review was undertaken using Pubmed database to collect articles evaluating directly or indiretly the impact of insecticide resistance and the associated mechanisms on key determinants of malaria vector competence including sialome composition, anti-*Plasmodium* immunity, intestinal commensal microbiota, and mosquito longevity. Globally, the evidence gathered is contradictory even though the insecticide resistant vectors seem to be more permissive to *Plasmodium* infections. The actual body of knowledge on key factors to vectorial competence, such as the immunity and microbiota communities of the insecticide resistant vector is still very insufficient to definitively infer on the epidemiological importance of these vectors against the susceptible counterparts. More studies are needed to fill important knowledge gaps that could help predicting malaria epidemiology in a context where the selection and spread of insecticide resistant vectors is ongoing.

## Background

Malaria is the biggest killer among vector-borne diseases [1] and has claimed the lives of milllions of people over centuries [2]. In 2020, 241 million cases were reported leading to 627,000 deaths. The African region has paid the highest tributes with 96% of all deaths [3]. Malaria disease is caused by *Plasmodium* parasites, which are transmitted to humans by the bites of infected female mosquitoes of the genus *Anopheles* [4]. In Africa, *Plasmodium falciparum* is the most epidemiologically important of malaria parasites infecting humans [5], and *Anopheles gambiae*, *Anopheles coluzzii*, *Anopheles funestus* and *Anopheles arabiensis* are the dominant vector species [6].

Malaria control includes medical treatment of cases and protective measures against the vectors to prevent and/or limit contacts with human hosts during which transmission occurs. The control of mosquito populations on a large scale using insecticide-treated nets (ITNs) and indoor residual spraying, associated with increase case management, has led to a remarkable reduction in malaria burden from 81.1 cases per 1000 population in 2000 to 58.9 in 2015 [3]. After this period, the impact of control efforts on malaria burden have dwindled, coinciding with the spread of insecticide resistant vectors across most endemic countries [3, 7]. Resistance of *Anopheles* mosquitoes to insecticides, reported for the first time in Africa in the 1950s [7], concerns four main classes of insecticides used in public health for vector control purposes, namely pyrethroids, organochlorines, organophosphates and carbamates [7, 8]. There are four mechanisms deployed by mosquitoes to become insensitive to the insecticides, including by order of importance (1) degradation of insecticide molecules by detoxification enzymes (metabolic resistance), (2) modification of the target affinity of the insecticide (target site resistance), (3) reduced penetration of the insecticide (cuticular resistance) and, (4) avoidance of insecticide-treated surfaces (behavioural resistance). Of these four mechanisms target site and metabolic resistances are most likely to lead to control failure [9].

In target site resistance, a change (leucine changed to a phenylalanine or a serine at position 1014) occurring in the amino acid sequence of the voltage gate sodium channel (vgsc) leads to a reduced sensitivity of mosquitoes to pyrethroids and organochlorines. This phenotype is known as knock down resistance or kdr [10, 11]. When the amino acid change (glycine replaced by serine at position 119) occurs in the neurotransmitter acetyl-cholinesterase, it occasions resistance to organophosphates and carbamates, termed ace-1 resistance [12, 13]. About metabolic resistance, insecticide resistant mosquitoes increase the expression of detoxification enzymes, such as the cytochrome P450 monooxygenases, glutathione S-transferases (GSTs) and esterases, that eliminates xenobiotic compounds (including insecticides) before they reach their target. In another instance, an amino acid substitutions in the sequence of detoxification enzymes could modifiy its affinity with the insecticides in insect vectors [14]. For example, several cytochrome P450 genes (*CYP6P9a*, *CYP6P9b* and *CYP6M7*) are involved in resistance to pyrethroids in the species *An. funestus* [15, 16]; while a substitution of leucine by phenylalanine at position 119 in the epsilon class of GST (*GST2*- L119F) confers a cross-resistance to dichloro-diphenyl-trichloroethane (DDT) and pyrethroids in the same vector species [17].

Despite the widespread distribution of insecticide resistance, its impact on overall malaria epidemiology remains unclear and is currently a subject of intense debate. The evaluation of the potential impact of insecticide resistance on vectorial competence is therefore becoming an important and urgent research theme whose findings will help understanding whether it alters or enhances the permissiveness of malaria vectors to *Plasmodium* parasites, from its early stage (ookinete) to the infective form (sporozoite). In this review, the evidence of insecticide resistance impact on the infectivity of mosquitoes to *Plasmodium* was explored in the literature, and changes in intrinsic factors that could predict or explain the outcome of an infectious blood meal intake were broached. Finally, the knowledge gaps were pointed out.

### Search strategy

A literature search was undertaken in the PubMed database to extract articles addressing the following themes: (1) *Plasmodium* infection in insecticide resistant malaria vectors, (2) sialome of insecticide resistant malaria vectors, (3) effect of insecticide resistance on the immunity of malaria vectors, (4) microbiota of insecticide resistant malaria vectors and infection, and (5) fitness cost of insecticide resistance in malaria vectors. The first search terms were “*Anopheles*” and “insecticide resistance” and they were associated with either “*Plasmodium* infection”, “vector competence”, “salivary gland”, “sialome”, “microbiota”, “gene expression” or “longevity”. Additional articles were extracted from the references lists of the full publications. The search was done between February and August 2022 and there was no restriction regarding the date of publication of the articles. A total of 560 articles were obtained from the search. Articles that addressed insecticide resistance in *Anopheles* in a broad manner, and not in relation with either *Plamodium* infection, vector competence, sialome, or longevity were discarded. Therefore, 28 articles related to the themes mentioned above were selected and used for the review.

### Malaria vector competence

Vector competence is the intrinsic ability of anopheline species or populations to allow the development of *Plasmodium* parasites from ookinete to infective sporozoites. When a mosquito takes an infectious blood meal from human, the gametocytes ingested begin their development in the midgut. The male gametocyte transform into eight microgametes after three rounds of mitosis, meanwhile the female gametocytes matures into macrogametes [4]. These cells fuse to form zygotes that thereafter change into ookinetes in the lumen of the intestine. The ookinetes then strive through the epithelium of the midgut and once in its basal side, transform into oocysts. The oocysts undergo several rounds of asexual multiplication (sporogony) leading to the production of thousands of haploid sporozoites in each oocyst. Mature occysts rupture and release sporozoites in the hemocoel, which immediately migrate to the salivary glands. The extrinsic incubation period of the parasite is about 14 days with the transition from ookinetes to mature oocysts having the highest duration (about 10 days) [18, 19].

**Fig. 1:**
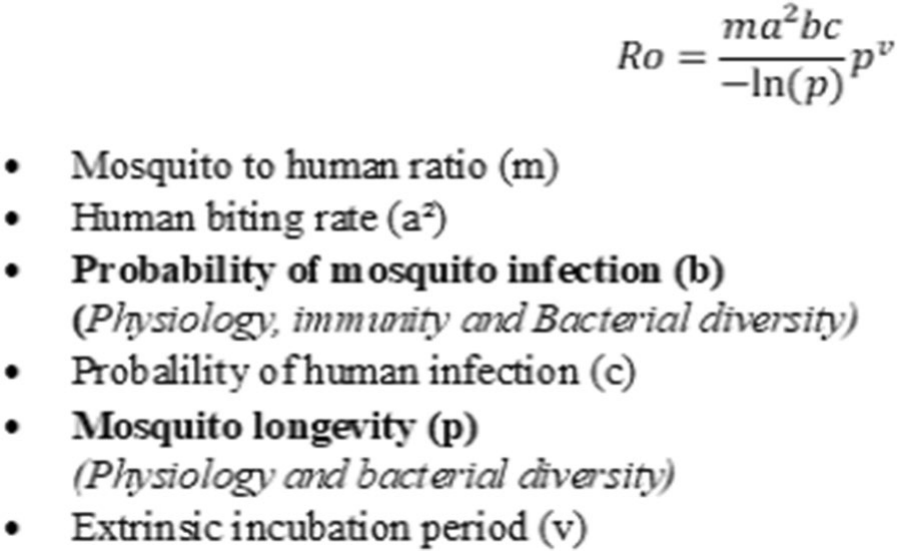
Basic reporductive number (Ro), Ross-MacDonald model. In bold, parameters of the vectorial competence influenced by insecticide resistance

In mosquito host, *Plasmodium* face several immune-related bottlenecks deployed to prevent the successful transition from its early stage in the midgut to the sporozoite stage in the salivary glands [18]. The outcome of the parasite infection is reported to depend mainly on the mosquito-*Plasmodium* genetics adaptation [19, 20]. Another very important factor that influences the above outcome is the compatibility of the duration of parasite development with the longevity of the mosquitoes [21, 22]. Only species in which *Plasmodium* reaches infective form are referred to as competent vectors and could ensure malaria transmission. The impact of vector competence on the transmission of malaria can be estimated using Ro (Fig. 1), the basic reproductive number developed by McDonald in 1957. The McDonald model gives the threshold for a disease to persist or spread (Ro greater than 1) or to disappear (Ro less than 1) [23]. The Ro represents the number of individuals in a susceptible human population that are expected to get infected via a mosquito bite when a single infected individual is present in the population [24, 25]. In the Ro equation, two parameters are related to vector competence: probability of mosquito infection (b) and mosquito longevity (p) (Fig. 1). Modifications of the values of components of this equation for a given vector population will cause either an augmentation or reduction in the transmission dynamics of the disease, leading probably to a change in the epidemiological profile of the locality concerned. It was established that an increase in b will increase the Ro, whereas a decrease in p will cause the opposite [26].

### Insecticide resistance and malaria vector infectivity to *Plasmodium* parasite

The rapid spread of insecticide resistance among malaria vectors accross endemic countries in the past decade have raised several questions among which that of knowing what is its impact on mosquito permissiveness to *Plasmodium*? Only a limited number of studies have tried to elucidate this question [27–35]. These studies compared *P. falciparum* infection rates in resistant Anopheline vectors with susceptible ones, either caught in the field or experimentally infected (Table 1).

**Table 1:** Summary of studies evaluating the impact of insecticide resistance on malaria vectors susceptibility to *P. falciparum* infection

Mosquito species	Type of experiment performed	Type of infection	Infection outcome		References
			Prevalence	Intensity	
*An. gambiae*^*a*^	*kdr*-resistant strain compared to susceptible strain	Natural	Higher sporozoite infection prevalence in resistant strain^s^		[27]
*An. funestus s.s*^*a*^	*GSTe2*-resistant genotypes compared to susceptible genotypes	Natural	No difference in oocysts infection prevalence ^ns^ Higher sporozoite infection prevalence in resistant strain^s^		[28]
*An. gambiae* s.l.^*a*^	*ace-1*-resistant strain compared to susceptible strain	Natural	Higher oocyst infection prevalence in resistant strain^s^		[29]
*An. gambiae* s.l.^*a*^	*kdr*-resistant strain compared to susceptible strain	Natural	No difference in oocysts infection prevalence ^ns^		[29]
*An. gambiae*^*a*^	*kdr*-resistant strain compared to susceptible strain	Experimental	Higher oocyst infection prevalence in resistant strain^s^ Higher sporozoite infection prevalence in resistant strain^s^	Higher oocyst load in the resistant strain^s^ Higher sporozoite infection load in the resistant strain^s^	[30]
*An. coluzzii*^*a*^	*kdr*-resistant strain compared to susceptible strain	Experimental	Higher oocyst prevalence in the resistant strain^s^ Higher sporozoite infection prevalence in resistant strain^s^	Higher oocysts load in the resistant strain^s^ Higher sporozoite load prevalence in the resistant strain^s^	[30]
*An. funestus s.s*^*a*^	*GSTe2*-resistant genotypes compared to susceptible genotypes	Experimental	Lower oocyst infection prevalence in homozygous resistant genotypes^s^	Higher oocysts load in homozygous and heterozygous resistant genotypes^s^	[31]
*An. gambiae*^*b*^	*ace-1*-resistant strain compared to susceptible strain	Experimental	Higher oocyst prevalence infection in resistant strain^s^ No difference in sporozoite infection prevalence^ns^	No difference in oocyst and sporozoite infection load^ns^	[32]
*An. gambiae*^*b*^	*kdr*-resistant strain compared to susceptible strain	Experimental	Higher oocyst infection prevalence in resistant strain^s^ Higher sporozoite infection prevalence in resistant strain^s^	Lower oocyst and sporozoite infection load in resistant strain^s^	[32]
*An. gambiae*^*b*^	*ace-1*-resistant strain compared to susceptible strain		Higher oocyst infection prevalence in resistant strain^s^		[33]
*An. gambiae*^*b*^	*kdr*-resistant strain compared to susceptible strain	Experimental	Higher oocyst infection prevalence in resistant strain^s^		[33]
*An. gambiae*^*b*^	*kdr*-resistant strain compared to *kdr*-resistant strain exposed to insecticides		Lower oocyst infection prevalence in resistant strain^s^	No difference in oocyst infection load^ns^	[34]
*An. gambiae*^*b*^	*ace-1*-resistant strain compared to *ace-1*-resistant strain exposed to insecticides	Experimental	Lower oocyst infection prevalence in resistant strain^s^	Lower oocyst infection load in resistant strain^s^	[34]
*An. gambiae*^*a*^	*kdr*-resistant strain compared to *kdr*-resistant strain exposed to insecticide-treated nets	Experimental	Lower oocyst infection prevalence in resistant strain^s^	Lower oocyst infection load in resistant strain^s^	[35]

a: field strain; b: laboratory strain; ns: Non Significant; s: Significant; α: insecticide exposure to confirm resistance status probably post infection; β: insecticide exposure to confirm resistance status prior to infection

*Anopheles gambiae* strain bearing *kdr* resistance allele (*Vgsc*-*L*1014S) was found naturally more infected by sporozoites than the susceptible counterpart [27]. Similar findings were experimentally observed in the same species, as well as in *Anopheles coluzzii* [30, 32]. Contrary to *kdr* resistance, *An. gambiae* with *ace-1* resistance allele did not differ from individuals that have the wild type allele (not conferring insecticide resistance) on infection rate despite significantly higher oocyst prevalences were observed in the resistant strain [32]. More studies using field populations are needed to ascertain whether a lower longevity suspected by the author and/or other factors are involved.

Regarding metabolic resistance, recent breakthroughs in designing simple PCR-based assays to detect glutathione S-transferase (GST)-based and cytochrome P450-mediated resistance in *An. funestus *sensu stricto provided a unique opportunity to assess its impact on the mosquito’s ability to develop the parasites. The L119F-*GSTe2* resistant genotypes of this species showed, in an experimental infection study, higher permissiveness to oocyst infections than susceptible ones [31]. Similarly, in naturally infected populations of the same species, homozygote L119F-*GSTe2* genotypes were found more infected by sporozoites though no significant difference was found at the level of oocyst prevalence [28]. In other hands, Lo and Coetzee [36], infecting experimentally two selected sub-colonies of FUMOZ displaying different degree of pyrethroid resistance by *Plasmodium berghei*, found that the insecticide resistant colonies were less permissive to infection than the susceptible ones. No investigation has so far explored the relationship between P450s genes implicated in insecticide resistance and *P. falciparum* infection in *An. funestus*. Moreover, because of the absence of markers of metabolic resistance in *An. gambiae *sensu lato such studies are still lacking in these species.

### Impact of insecticide resistance on mosquito sialome

Bloodsucking arthropods, like mosquitoes, have evolved saliva containing a mixture of pharmacologically active molecules that help them counteract the hemostatis and inflammatory responses of the vertebrate host during bites, thus facilitating blood meal intake [37]. However, the activity of these molecules goes beyond the scope of ensuring blood meal success, as they possibly influence the completion of *Plasmodium* development in the salivary gland of malaria vectors. Proteins secreted by the salivary gland belong to several families (D7, mucin, gSG1, gSG2, gSG6 peptide, gSG7, cE5, 8.2-kDa, 6.2-kDa, etc.) [38] whose function include (1) cytoskeletal and structural activities (2) digestion, (3) circadian rythm and chemosensory, (3) immunity, (4) metabolism and other [39]. The development of insecticide resistance in malaria vectors is accompanied by physiological changes [26] that may affect the sialome composition with consequences on the vector competence. Few studies have investigated changes in the sialome in the insecticide resistant vectors [40, 41].

The secretory protein 100 kDa, which is encoded by *Saglin* (a cytoskeletal and structural gene present in *An. gambiae* salivary gland) was considered as the binding target of *P. falciparum* and *P. berghei* on salivary gland prior to penetration into the latter [42]. This protein was found down-regulated in *ace-1* bearing *An. gambiae* strain, suggesting an impact on the vector infectivity to *Plasmodium* [43]. However, a recent study showed that the 100 kDa Protein is unevenly distributed on the salivary glands lobes. Its absence on the primary site of sporozoites occupancy in the salivary glands, the distal lateral lobes, implies that this protein may instead have a secondary role in the infection of the organ [44–46].

The D7 salivary family has been identified in malaria vectors among the most expressed proteins involved in the antihemostatic activity and probably in digestion of blood meal [47–50]. Elanga et al. [40] showed that two short forms of the D7 family genes (*D7r3* and *D7r4*) are over-expressed in pyrethroid resistant *An. funestus* (*L119F*-*GSTe2*), whereas almost all D7 genes are under-expressed in pyrethroid resistant *An. gambiae* (*kdr*, *L1014F*). A comparable observation was made in insecticide resistant *Culex quinquefasciatus* (*ace-1* resistance) [51] as well as in two strains of *Aedes aegypti* (homozygotes resistant *C1534* and *G1016 kdr*) [52]. These findings show that insecticide resistance mechanism may affect the sialome composition differently.

Several immune proteins such as the anti-microbial peptides cecropin and defensin were found in the saliva of mosquitoes [39, 53]. These immune proteins underscore the role of the salivary gland in the refractoriness of the *Anopheles* to infections [39, 53]. The small number of studies that evaluated the impact of insecticide resistance alleles on salivary gland gene expression in mosquito vectors have not reported significant changes related to immune genes as compared with the susceptible counterparts [41, 43, 51, 52], alluding that the resistant status to insecticide does not influence noticeably the immune component of the sialome. If these factors are indeed unchanged regardless of the mosquito allelic composition, nothing is known whether under infection the expression profile of these immune proteins will vary or not according to the mosquito genotype. Das et al. [39] and Djegbe et al. [51] demonstrated that salivary gland genes expression is influenced by blood meal intake and varies towards the period coinciding with the maturation of *Plasmodium* parasites in mosquitoes [54]. This evidence was not previously studied and should be taken into account in subsequent research works that aims at identifying differentially expressed genes of the salivary gland and elucidating their impact on the malaria vector competence.

### Impact of insecticide resistance on vector immunity

When the infectious blood meal reaches the midgut of the female *Anopheles*, the immune system is deployed to prevent infections [20]. In the midgut, *P. falciparum* faces the peritrophic membrane, a physical barrier developed to prevent infections. It also protects against the damaging effects of the human blood factors like antibodies and regulates several digestive enzymes [55, 56]. Enzymes such as trypsin 1 and 2, chymotrypsin, carboxypeptidase, aminopeptidase and serine protease are upregulated during digestion to cleave the large content of proteins in the blood meal [57–60]. These proteases are apparently involved in the elimination of *Plasmodium* infections [61]. Three studies attempted to elucidate the effect of insecticide resistance on vectors’ immunity [62–64]. Mitri et al. [62], in a study evaluating genes implicated in the infectivity of *An. coluzzii*, demonstrated that the *kdr*-bearing para gene which carries mutations of the voltage-gate sodium channel (confering insecticide resistance) is not associated with infection but rather the *ClipC9* gene directing the synthesis of Serine protease. This suggest that the effect of the resistant character on refractoriness to infection may be due to genes other than that involved in resistance to insecticides, and which happen to be linked to it. The Serine protease plays an important role in the activation of the three major immune signaling pathways in mosquitoes: Toll, Imd and JAK/STAT [20], which cause the release of antimicrobial peptides (AMPs) notably defensins, cecropins, attacin, gambicin and *AgSTAT-A,* effective against malaria parasites infections. Vontas et al. [63], using pyrethroid and organochlorine resistant *An. gambiae* strains, showed that defensin and cecropin are upregulated after pre-exposure to permethrin. This study sugggests that insecticide resistant mosquitoes may be better equipped than susceptible ones to combat infections, but these two immune effectors alone may not be decisive in rendering the vector completely refractory to malaria infections as many other pathways activated concomitantly during parasitic invasion are altogether implicated in the outcome of a contamination [20].

In *Culex pipiens* which is vector of many pathogens including arboviruses [65], filarial worms [66], and protozoa [67], immune response was stimulated in an insecticide resistant field strain by injection of Lipopolysacharide (LPS) immune elicitor. As result, no difference was found in the expression of defensin and cecropin as compared to the control group; but only an increase in gambicin was recorded [68]. One point can be drawn from these results to infer what might happen in malaria vectors: *Plasmodium* infections may trigger the overexpresion of some immune factors while the other may have their expression either down regulated or unchanged.

The reactive oxygen species (ROS) produced by cellular metabolism are another class of effectors of the innate immunity that can negatively affect malaria parasites [69, 70]. They kill the parasites through both lytic and melanization pathways [20]. Ingaham et al. [64] showed that *An. coluzzii* VK7 colony displaying *kdr* resistance mechanism, *CYP6M6* and *CYP6P3* metabolisers, had oxidoreductase overexpressed after sub-lethal exposure to deltamethrin, suggesting that this species could be more refractory to *Plasmodium* infection. At this point, it is necessary to verify whether under natural conditions, insecticide-resistant *Anopheles* mosquitoes will display an overexpression of ROS or not.

Cellular immune responses are carried out by varous type of hemocytes that eliminate pathogens by phagocytosis, lysis and melanization [20]. Organochlorines and organophosphate were found to affect differently the hemocytes abundance including granulocytes in the insect *Rhynocoris kumarii* [71]. In mosquitoes, studies are needed to ascertain the impact of insecticide resistance on cellular immunity and the resulting effect on the infectivity of resistant vector to malaria parasite. Regarding melanization of pathogens, it is lead by the phenoloxidase (PO) produced by Oenocytoids [72, 73] and is regulated by serine protease inhibitors. In field-caught *C. pipiens* resistant to insecticide through an increase in detoxification (esterase) and target site mutation (ace-1), PO expression was equal to that of susceptible group [74], suggesting that some genes associated with immunity might not be affected by insecticide resistance character in mosquitoes. No studies have verified the effect of insecticide resistance on PO in malaria vectors.

### Impact of insecticide resistance on commensal intestinal microbiota of malaria vectors

Bacteria, fungi and viruses colonize the gut, salivary glands and reproductive organs of the mosquitoes. These microorganisms are mainly acquired from the environnement and its composition is largely influenced by its aquatic breeding sites [75, 76]. In addition, the microbiota composition is highly dynamic, varying greatly with localities and seasons [77–79]. These variations of microbiota composition within field mosquitoes may partly explain the variability in infection levels in the field [80].

Mosquito microbiota has great potential for impeding the transmission of malaria by altering vectorial capacity [81]. Also, the microbiota is capable of influencing the biology of the host such as altering its immunity, nutrition, digestion, vectorial competence, reproduction, and insecticide resistance [82–87]. With the growing concerns about the rapid spread of insecticide resistance in *Anopheles* mosquitoes, some studies have explored the functions of the mosquito's gut microbial communities in the development of resistance. For example, distinct microbita populations were found associated with organochlorine resistance in *An. arabiensis* [86] and *Anopheles albimanus* [88]. Similarly, an association between specific microbiota and intense pyrethroid resistance was reported in *An. gambiae* [89] and *Anopheles stephensi* [90], suggesting a microbiota-mediated insecticide resistance mechanism. Dieme et al. [91] suggested that changes in the feeding behaviour of insecticide resistant vectors may lead to higher microbial diversity. This diversity could modify the repertoire of protective bacteria against pathogen infections and/or that of their enhancers, with consequences on the vectorial competence [9, 92]. Recently, Bassene et al. [93] showed that, in the species *An. gambiae* and *An. funestus*, the microbiota was signifanctly different between *P. falciparum*-infected and non-infected samples, although the resistance status of these mosquitoes was not evaluated. More refined studies are needed to characterize the microbial communities harboured by the insecticide resistant malaria vectors. Also the contribution of microbiota against other factors to the vectorial competence of insecticide resistant malaria vectors remains to be investigated.

### Impact of insecticide resistance on the longevity of malaria vectors

Mosquito longevity is a determinant factor for parasite maturation and could influences malaria transmission [94, 95]. In fact, the extrinsic incubation of *Plasmodium* in its hematophageous host is about 11–14 days. Therefore, only mosquitoes whose lifespan is long enough could allow the complete development of the malaria parasite to the sporozoite infective stage and participate in the transmission of the disease. With the emergence and spread of insecticide resistance [7], many investigations were undertaken to gain knowledge of the effect of this phenomenon on the vectors’ longevity and so on its potential epidemiological impact.

So far, studies on the impact of insecticide resistance on malaria vectors longevity have focused on four species: *An. gambiae, An. arabiensis, An. coluzzii* and *An. funestus*. Globally, the findings revealed a pleitropic effect of insecticide resistance on mosquito lifespan [33, 96–108] (Table 2). The majority of studies (10/14) which used laboratory strains showed that pyrethroid resistant *An. funestus* and *An. gambiae* live longer than susceptible ones [100, 106]. Of the studies including field strains, a longer life span was reported in organochlorine and pyrethroid resistant *An. funestus* strains [104, 105]. In contrast, a shorter life span was observed in *An. gambiae* strain resistant to organochlorine. It was reported that pre-exposure to insecticide in a manner micmicing field exposure to insecticides, affects the longevity of insecticide resistant *An. gambiae* strain [97], and that delayed mortality observed in the vectors may be dependent on resistance intensity [98]. This later observation indicates that findings obtained with laboratory colonies are to be taken with caution given that they may not reflect exactly what is oberved on the field [26]. Nevertheless, such studies remain important as they contribute to the understanding of the potential mechanisms affecting the vectors’ longevity [109, 110], notably resource-based trade-off and oxidative stress.

**Table 2:** Summary of studies assessing the impact of insecticide resistance on the longevity of malaria vectors

Study species	Origin of strains	Class of insecticides	Resistance mechanism(s)	Pre-Exposure^a^/Exposure^b^ to insecticide	Effect on longevity	References
*An. gambiae s.l*	Field	PY	Not available	Deltamethrin and permethrin^a^	RR longevity > SS longevity	[97]
	Field	PY & OC	*kdr*	Not applied	RR longevity < SS longevity	[96]
	Field	PY	Not available	Permethrin (Net) ^b^	RR^e^ longevity = RR^ne^	[98]
	Laboratory	PY & OC	*kdr* & P450	Permethrin (Hut-net) ^b^		
	Laboratory	PY	Not available	Deltamethrin^b^	RR longevity < SS longevity	[99]
	Laboratory	PY	kdr, P450 and esterase	Not applied	RR longevity > SS longevity	[100]
	Laboratory	OC	*kdr*	DDT^a^	RR longevity = SS longevity	[33]
		CA	*ace*-1	Bendiocarb^a^	RR longevity > SS longevity	
	Laboratory	OC	*kdr*, *GSTe*, P450 & Es	Not applied	RR longevity = SS longevity	[101]
	Laboratory	OC	*GSTe,* P450 & Es	Not applied	RR^b^ longevity > RR^s^	[102]
	Laboratory	OC	*GSTe*, P450, Es;*kdr*	Not applied	RR longevity < SS longevity	[103]
				DDT^a^	RR^e^ longevity = RR^ne^	
				Permethrin^a^,	RR^e^ longevity < RR^ne^	
				Deltamethrin^a^	RR^e^ longevity < RR^ne^	
				Malathion	RR^e^ longevity < RR^ne^	
*An. funestus*	Field	OC	*GSTe2*	Permethrin (Net) ^b^	RR longevity > SS longevity	[104]
	Field	PY & OC	*GSTe2*	Not applied	RR longevity > SS longevity	[105]
	Laboratory	PY	Not available	Not applied	RR longevity > SS longevity	[106]
	Laboratory	PY & CA	*P450-a*	Not applied	RR longevity = SS longevity	[107]
	Laboratory	PY & CA	*P450-a*	Not applied	RR longevity = SS longevity	[108]
			*P450-b*		RR longevity = SS longevity	
			*P450-a/P450-b*		RR longevity = SS longevity	

PY: Pyrethroid; OC: Organochlorine; CA: Carbamate; GSTe: Glutathion S-transferase; Es: Esterase; *kdr*: Knock down resistance; P450: Cytochrome P450 monoxygenase; RR: resistant strain, SS: susceptible strain; RR^e^: resistant strain exposed to insecticide; RR^ne^: resistant strain non-exposed to insecticide; RR^b^: Resistant strain fed on blood; RR^s^: Resistant strain fed on sugar; > more; < less; = equal

Resource based trade-off is an evolutionary ecology concept that states that when environmental constraints lead to the augmentation of resources to one biological trait, other traits will have their energy budget reduced [111]. Accordingly, when mosquitoes adopt the detoxification mechanism to prevent the effect of insecticide, an increased production of detoxifying enzymes follows and is maintained by the additional resources deployed for the function. Otali et al. [100] have demonstrated that metabolism and longevity of insecticide resistant *An. gambiae* are lower than that of the susceptible strain. Moreover, they showed that the resistant strain has higher Reactive Oxygen Species (ROS), which are factors determining oxydative stress. In fact, the ROS are multifunctional molecules produced by cells of all organisms during normal metabolism [112, 113]. They have been pointed out as key aging factor in other organisms including *Anopheles* [114]. Therefore, mosquitoes that develop the capacity to cope with oxydative stress are likely to live longer. Oliver and Brooke [103] in an experiment evaluating the effect of oxidative stress on the longevity of both *An. arabiensis* and *An. funestus* bearing respectively *kdr* and Cytochrome P450 mechanisms demonstrated that these species live longer, and that Cytochrome P450 activity seems more protective against oxydative stress.

Rivero et al. [26] proposed the potential effect of different detoxifying enzymes on vector longevity. For example, Glutathion S-Transferase is considered to protect against oxydative stress. Confirming this point, a longer lifespan implicating Glutathion S-Tranferase in *An. funestus* was revealed with and without exposure to insecticide [104, 105]. In contrast, monoxygenase, known to be associated with an increase in oxydative stress has not led, as expected, to reduced longevity in *An. funestus* [108]. More studies using field populations and micmicing field conditions are necessary to ascertain the full impact of insecticide resistance on longevity of the malaria vector.

## Conclusion

The need for a comprehensive understanding of the impact of insecticide resistance on malaria vector competence is unquestionable. The current state of knowledge is not only insufficient but also contradictory to draw a definitive conclusion. A tendency nevertheless emerges from findings that insecticide resistance may increases the infectivity of malaria vectors to *Plasmodium*, thus their vector competence. This is possibly due to changes in the expression of some genes notably those involved in blood-feeding and the immunity. Additionally, microbiota communities vary in the resistant mosquitoes as compared to the susceptible counterparts. The actual effect of these changes in the course of infection and their impact on the infectivity of malaria vectors to *P. falciparum* is still to be investigated. Finally, the longevity of the vectors is not always affected by insecticide resistance mechanisms. It is worth noting that, studies using vectors displaying metabolic resistance were under-represented because molecular markers to diagnose this character were developped only recently, especially in *An. funestus*. Malaria vectors that bear metabolic resistant mechanism are, on an ecological immunology point of view, expected to have a number of biological functions impaired, including immunity. If established, this situation may cause them to become less refractory to *Plasmodium* infection. Taking advantage of recent advances in the genomics, transcriptomics and molecular characterization of insecticide resistance, more refined studies can now be undertaken to fill knowledge gaps regarding the effect of insectide resistance on key determinants of vectorial competence and subsequently predict changes in the epidemiology of malaria in a context of insecticide resistance escalation.

## Data Availability

Not applicable.

## Abbreviations

DDT: Dichloro-diphenyl-trichloroethane
GST: Glutathione S-transferases
kdr: Knock down resistance
LPS: Lipopolysacharide
PO: Phenoloxidase
Vgsc: Voltage gate sodium channel
